# Study of the Cu(111) Surface by Scanning Tunneling Microscopy: The Morphology Evolution, Reconstructions, Superstructures and Line Defects

**DOI:** 10.3390/nano12234278

**Published:** 2022-12-01

**Authors:** Zhaochen Qu, Xiaodan Wang, Xiangqian Shen, Hua Zhou

**Affiliations:** 1 School of Physics, Shandong University, Jinan 250100, China; 2 Engineering Research Center of Micro-Nano Optoelectronic Materials and Devices, Ministry of Education, Fujian Key Laboratory of Semiconductor Materials and Applications, CI Center for OSED, and Department of Physics, Xiamen University, Xiamen 361005, China; 3 School of Physics and Technology, Xinjiang University, Urumqi 830046, China

**Keywords:** scanning tunneling microscopy, Cu(111) surface, morphology evolution, surface superstructures, reconstruction

## Abstract

The Cu(111) surface is an important substrate for catalysis and the growth of 2D materials, but a comprehensive understanding of the preparation and formation of well-ordered and atomically clean Cu(111) surfaces is still lacking. In this work, the morphology and structure changes of the Cu(111) surface after treatment by ion bombardment and annealing with a temperature range of 300–720 °C are investigated systematically by using in situ low-temperature scanning tunneling microscopy. With the increase of annealing temperature, the surface morphology changes from corrugation to straight edge, the number of screw dislocations changes from none to numerous, and the surface atomic structure changes from disordered to ordered structures (with many reconstructions). In addition, the changing trend of step width and step height in different stages is different (first increased and then decreased). A perfect Cu(111) surface with a step height of one atom layer (0.21 nm) and a width of more than 150 nm was obtained. In addition, two interesting superstructures and a new surface phase with a large number of line defects were found. This work serves as a strong foundation for understanding the properties of Cu(111) surface, and it also provides important guidance for the effective pretreatment of Cu(111) substrates, which are widely used.

## 1. Introduction

As one of the most important transition metal elements, Copper (Cu) is a basic material of modern technology. It has been applied in many fields, such as transmission wires, electrodes, electrocatalysts, thermoelectrics, IC industry, nanotheranostics, etc. [1,2,3,4,5]. Due to the important role of surface structures and properties in the preparation and application of Cu-based materials, research on Cu-based surfaces has attracted extensive attention in recent years. In particular, the Cu(111) surface is considered to be superior to other surfaces and has a strong catalyst activation ability as well as broad application prospects in catalysts [6,7]. For instance, more effective elimination of CO and NO and methanol synthesis via catalytic oxidation might be achieved by using Cu(111)-based catalysis [8,9,10,11,12]. In addition, the Cu(111) surface plays an important role in the study of basic surface science. For example, it supplies a platform for the study of standing wave electrons [13,14] and Kondo resonance [15]. 

Moreover, the Cu(111) surface fosters the development of 2D materials (which have potential applications in future electronics, optoelectronic devices, and quantum engineering, and have become a hot topic in recent decades [16,17,18,19]), and is considered an excellent and promising substrate in this field. For one thing, the use of it as a substrate might overcome the challenge of preparing 2D materials with large scale and high quality because it can meet the following three conditions as a valuable substrate: (1) it should be low-cost enough; (2) it should possess high temperature resistance; and (3) its surface structure should be fairly stable in air and have an inherent hexagonal character like most 2D materials. This has been experimentally confirmed in several 2D materials such as graphene and MoS_2_ [20,21,22,23,24]. In addition, although it is easy to obtain ideal 2D materials, superstructure and regular line defects are frequently observed in the surfaces of 2D materials, and their formation mechanisms are still poorly understood [25,26]. The morphology and structural characteristics of Cu(111) surface are important for understanding the structure and properties of 2D materials because of the close association between the surface structures and properties of 2D materials and the surface structures of the substrates. 

However, a comprehensive and accurate understanding of the preparation and formation mechanism of clean Cu(111) surfaces with atomically ordered structures is still lacking. In this work, an in-depth study on the surface structures of Cu(111) was carried out and the morphology and surface structure evolution of Cu(111) under treatments of different annealing temperatures were systematically studied by in situ low-temperature scanning tunneling microscopy (STM) technology (a powerful and useful tool having advantages in surface science research). The STM results showed that the step width and height first increased and then decreased with the annealing temperature ranging from 300 °C to 720 °C. A perfect surface with a step height of a single atom layer and a step width of more than 150 nm was successfully prepared. Moreover, two types of superstructures on the Cu(111) surface, consisting of circular clusters and double atoms, respectively, as well as a new surface phase with lots of line defects, were also observed. The present study laid the foundation for the further study of Cu(111) surfaces and their potential and broad use as valuable substrates. 

## 2. Materials and Methods

### 2.1. Sample Preparation

The Cu(111) single crystal sample with a size of 3 mm× 10 mm was commercially purchased from Hefei Kejing Material Technology Co., LTD. The whole experiment was carried out in a contacted ultra-high vacuum system consisting of a low-temperature scanning tunneling microscope (LT-STM) system (Createc, Germany), with a base vacuum level of better than 1 × 10^−10^ mbar and a preparation chamber under ultrahigh vacuum (UHV) conditions (base pressure ~2 × 10^−10^ mbar). The sample was first degassed at 250 °C with a vacuum level of less than 5 × 10^−9^ mbar for 30 min and then cleaned by Ar-ion bombardment with a kinetic energy of 1 keV and an argon pressure of 3 × 10^−6^ mbar. Annealing temperatures range from 300 °C to 760 °C. At lower temperatures less than 400 °C, the annealing time was 30 min, while at higher temperatures (400–760 °C), the annealing time was 3–10 min. The sample temperature was measured by an infrared radiation thermometer (Dikai Optoelectronics, Wuhan, China). After the annealing, the sample was first transferred to the LT-STM chamber for surface characterization by the STM and then transferred to the preparation chamber for the subsequent treatment. A series of treatment experiments were carried out in order in ten steps with corresponding conditions named “Process I”, “Process Ⅱ”, “Process Ⅲ”, “Process Ⅳ”, “Process Ⅴ”, “Process Ⅵ”, “Process Ⅶ”, “Process Ⅷ”, “Process Ⅸ”, and “Process Ⅹ”, respectively, with each consisting of 1–3 periods at the appropriate conditions, as illustrated in detail in Table 1. The vacuum level of the preparation chamber was maintained at better than 5 × 10^−8^ mbar during all annealing treatments. 

### 2.2. In Situ STM Characterization

All the STM images were obtained at 77 K in constant-current mode in the UHV analysis chamber. The bias voltage applied on the sample and the current were set at 0.5–2.0 V and 0.1 nA, respectively, and the scanning tip was grounded. The STM tip was prepared by cutting a Pt/Ir metal wire with a diameter of 0.2 mm and then sputtered by an electron beam with a kinetic energy of 600 eV. All the STM images were independently analyzed with the STMATM software (Version: 4.3.0.0, Createc, Germany).

## 3. Experimental Results

### 3.1. Morphology Evolution

**Figure 1:**
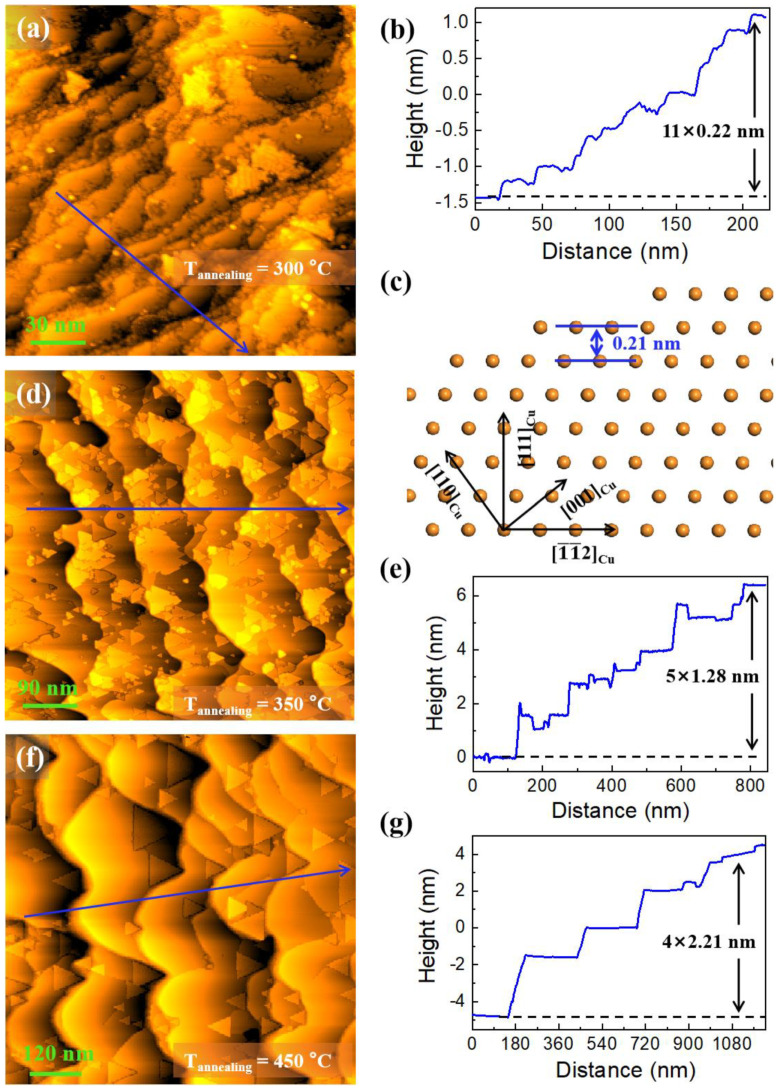
Morphology evolution from 300 °C to 450 °C. (**a**) STM image from the sample after Process Ⅰ annealing treatment, the blue arrow indicates the position where height profile was extracted. (**b**) height profile along the blue arrow in (**a**); (**c**) side view of the atomic model along the [1$\bar{1}$0]Cu azimuth; (**d**,**f**) the STM images from the sample after Process Ⅱ and Ⅲ annealing treatments, respectively, the blue arrows indicate the positions where height profiles were extracted; (**e**,**g**) height profiles along the blue arrows in (**d**,**f**), respectively. Here, the scanning bias voltage and current are equal to 1 V and 0.1 nA, respectively.

Figure 1a displays an STM image of the sample after the Process Ⅰ annealing treatment (at 300 °C), demonstrating the presence of steps with narrow widths and irregular edges at the sample surface. The average height of the steps is 0.22 nm, as illustrated in the line profile [drawn along the blue arrrow in Figure 1a] of Figure 1b. Theoretically, the minimum spacing (denoted by d) of planes along [111]_Cu_ is 0.21 nm [27], as indicated by the blue double arrow in the atomic structural model in side views [Figure 1c]. These results suggest that the steps at this stage are of only one atomic layer in thickness. After the Process Ⅱ annealing treatment (at 350 °C), the step width becomes wider while the step edge is still irregular, as shown in Figure 1d. Interestingly, many triangular-shaped islands are observed on the step terraces, with some overlapping regions. The height profile [drawn along the blue arrrow in Figure 1d] in Figure 1e shows that the average step height becomes 1.28 nm (≈6d). When the annealing temperature is raised to 450 °C (Process Ⅲ), the number of islands is significantly reduced, and the step width further increases, and more importantly, the boundary contour of the step edge looks like waves, as shown in Figure 1f. The line profile [drawn along the blue arrrow in Figure 1f] in Figure 1g shows that the average step height becomes 2.21 nm (≈10d). The evolution in the morphologies from Process Ⅰ to Ⅲ illustrates that the edges of the atomic steps become smoother and the steps become larger in height and width with the increase of annealing temperature.

**Figure 2:**
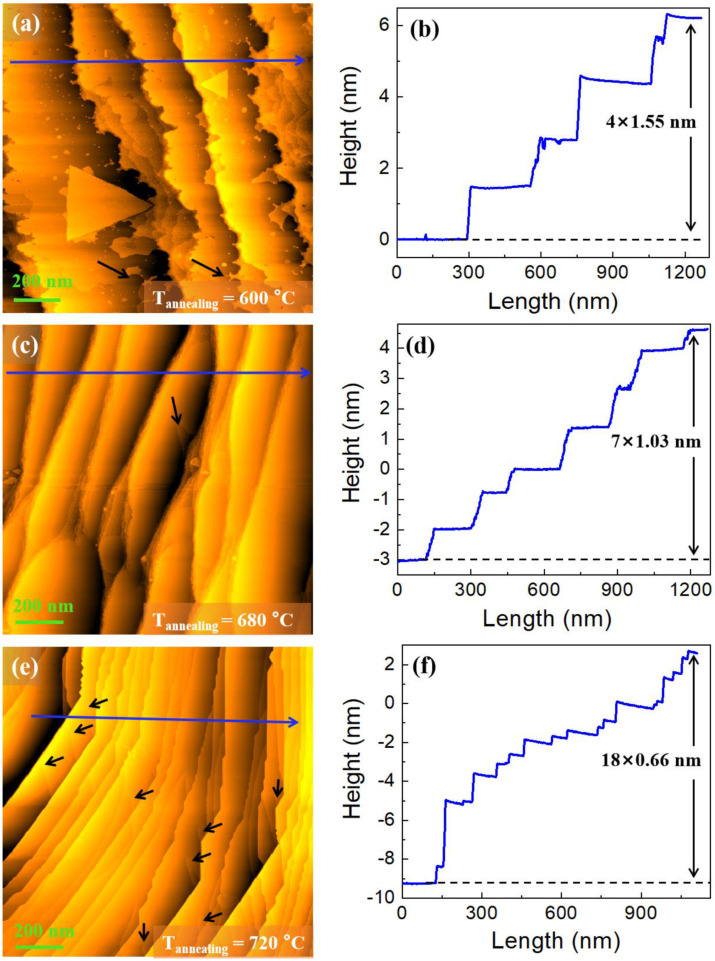
Morphology evolution from Process Ⅳ to Process Ⅵ. (**a**,**c**,**e**) STM images from the sample after annealing at 600 °C, 650 °C, and 720 °C, respectively, where the black arrows indicate the screw dislocations; (**b**,**d**,**f**) height profiles along the blue arrows in (**a**,**c**,**e**), respectively. Here, the scanning bias voltage and current are equal to 1 V and 0.1 nA, respectively.

When the annealing temperature is further raised to 600 °C (Process Ⅳ), zigzag edges are observed on the steps, and it presents a drastic reduction in the number of triangular islands as well as the appearance of a small number of screw dislocations (labeled by the black arrows) on the step terraces, as shown in Figure 2a. However, the average step height no longer increases but actually shows a decrease, with the value of 1.24 nm (≈6d) in this stage as shown in the height profile [drawn along the blue arrow in Figure 2a] in Figure 2b. When the annealing temperature reaches 650 °C (Process Ⅴ), straight edges are observed on the steps, the triangular islands on the step terrace disappear, and the screw dislocation remains observable (labeled by the black arrows), as shown in Figure 2c. The corresponding height profile in Figure 2d drawn along the blue arrow in Figure 2c shows a value of 1.03 nm (≈5d) for the step height, indicating that the steps continue to be numerically lower in the average height. When the annealing temperature reaches 720 °C (Process Ⅵ), it can be seen that straight edges are still observable on some steps and the other steps present sawtooth-like edges, with the step terraces becoming very smooth, as shown in Figure 2e. The average step height decreases to 0.66 nm (≈3d), as illustrated by the height profile [drawn along the blue arrow in Figure 2e] in Figure 2f. Interestingly, the step terraces show numerous screw dislocations in this process, as indicated by the black arrows in Figure 2e. This phenomenon has also previously been observed on other cleaned metal surfaces such as Au(111) surface, Ag(111) surface, etc. [28,29,30].

**Figure 3:**
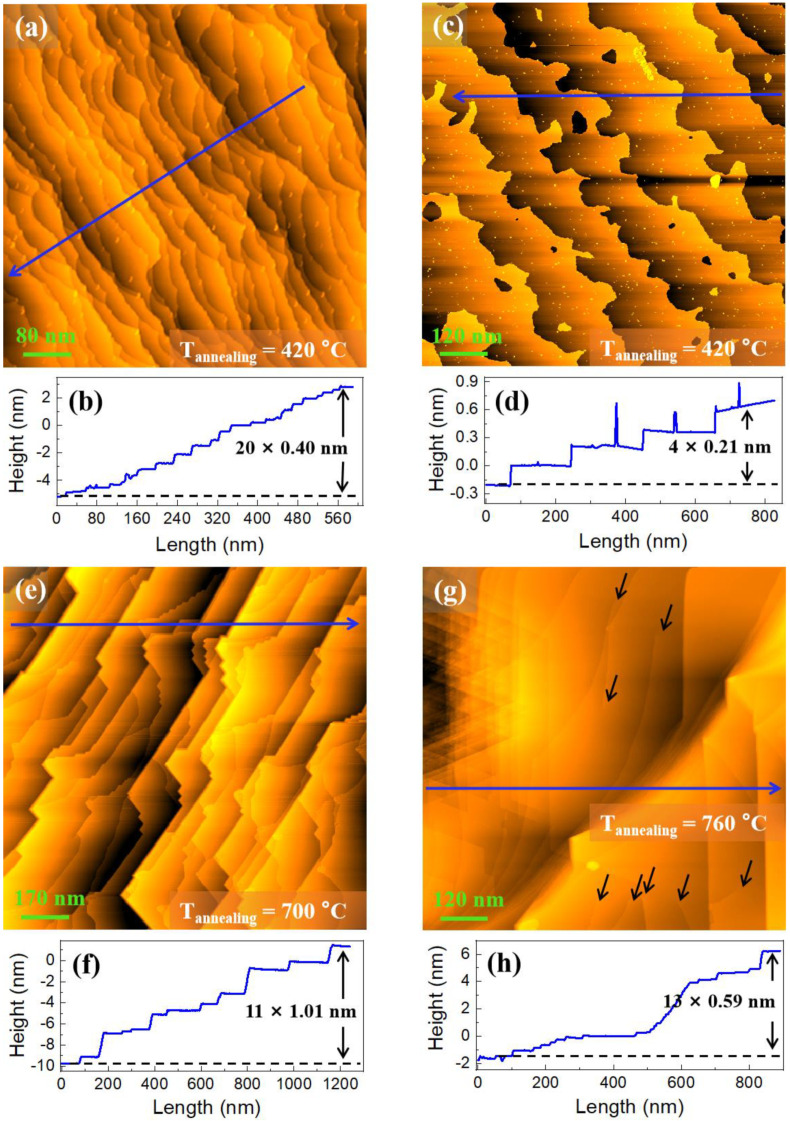
Morphology evolution from Process Ⅶ to Process Ⅹ. (**a**,**c**,**e**,**g**) STM images from the samples after annealing at 420 °C, 420 °C, 700 °C, and 760 °C, respectively; (**b**,**d**,**f**,**h**) the height profiles along the blue arrows in (**a**,**c**,**e**,**g**), respectively. The black arrows indicate the screw dislocations. Here, the scanning bias voltage and current in images (**a**,**b**) are equal to 1 V and 0.1 nA, and in images (**e**,**g**) are equal to 2 V and 0.1 nA, respectively.

To obtain a well-ordered and atomically clean Cu(111) surface, the sample was treated at the next lower temperature of 420 °C. Figure 3a shows the surface morphology of the sample after the Process Ⅶ treatment, and the wavy-like step edges can be seen. According to the height profile [drawn along the blue arrow in Figure 3a] in Figure 3b, the step width (height) is calculated to be about 28.5 nm (0.4 nm, ≈2d), which is far smaller (lower) than that in previous processes except for Process I (at 300 °C) shown in Figure 1a. The annealing temperature was maintained at 420 °C and the annealing time was changed to 10 min at the next process (Process Ⅷ). After this annealing treatment, we obtained a smooth surface with atomically flat terraces, large step width (more than 150 nm), and small step height (0.21 nm, = d, one atomic layer thick), shown in Figure 3c,d. These results indicate that well-ordered and atomically clean Cu(111) surface can be successfully obtained during the annealing process by first gradually increasing and then decreasing the temperature if the highest and the last lower temperatures are set at 720 °C and 420 °C, respectively.

When the annealing temperature reaches 700 °C, it can be clearly seen that the step edge becomes straight again and the average step height becomes 1.01 nm (≈5d), which instead significantly increased, as illustrated both by the STM image in Figure 3e and the height profile in Figure 3f. When the annealing temperature is further raised to 760 °C, the average step height becomes 0.59 nm, as illustrated by the height profile in Figure 3h, which begins to decrease again, but the STM image of the sample surface [Figure 3g] presents non-uniform characteristics. Simultaneously, many screw dislocations are observed on the terraces as indicated by the black arrows in Figure 3h. From the evolution of the surface morphology [as shown in the changes of the figures: Figure 2a → Figure 2c → Figure 2e → Figure 3c → Figure 3e → Figure 3h], we speculate that higher annealing temperature tends to induce more screw dislocations and cause the disappearance of surface step uniformity.

**Figure 4:**
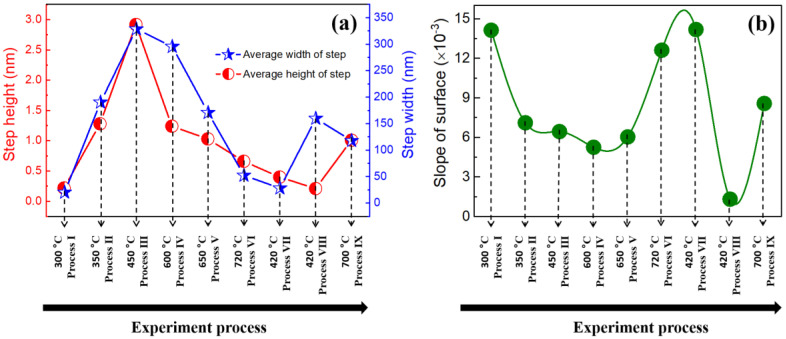
Step height, step width, and “surface slope” of the sample surface. (**a**) The changing dependence of average step width and height as a function of annealing process from Process Ⅰ (300 °C) to Process Ⅸ (700 °C); (**b**) the changing dependence of “surface slope” as a function of annealing process from Process Ⅰ (300 °C) to Process Ⅸ (700 °C).

To better understand the evolution of the surface structure, we plot the graph to illustrate the changing dependence of average step width and height as a function of annealing process, as shown in Figure 4a. It can be clearly seen that the average step height (width) first increases from 0.22 nm (17.6 nm) to 2.92 nm (343.8 nm) upon increasing annealing temperature from 300 °C (Process Ⅰ) to 450 °C (Process Ⅲ), and then decreases to 0.66 nm (52.3 nm) upon further increasing the annealing temperature to 720 °C (Process Ⅵ). Here, we define a quantity named “surface slope”, which is obtained by dividing the average step height by the average step width and can be regarded as an indicator of the surface roughness. The values of the “surface slope” have been calculated according to the data from Figure 1, Figure 2 and Figure 3, and their changes with annealing process variables are drawn in curves, as shown in Figure 4b. It shows that the “surface slope” first decreases and then increases when changing the annealing temperature from 300 °C (Process Ⅰ) to 720 °C (Process Ⅵ). In addition, as the annealing process changes from Process Ⅵ to Ⅸ (720 °C → 420 °C → 420 °C → 700 °C), the “surface slope” tends to first increase and then decrease. These results indicate that high-temperature annealing (>600 °C) could enhance surface roughness. The possible reason accounting for this result would be that the phenomenon of atomic desorption at the step edge becomes more pronounced when the annealing temperature is higher than 600 °C, and hence the step width becomes narrower but the step height becomes larger, leading to a larger “surface slope”. This phenomenon, where higher annealing temperature causes rougher and higher-step-density surfaces, has also been observed on other materials such as ZnO(0001) surface [31].

### 3.2. Reconstructions and Superstructures

**Figure 5:**
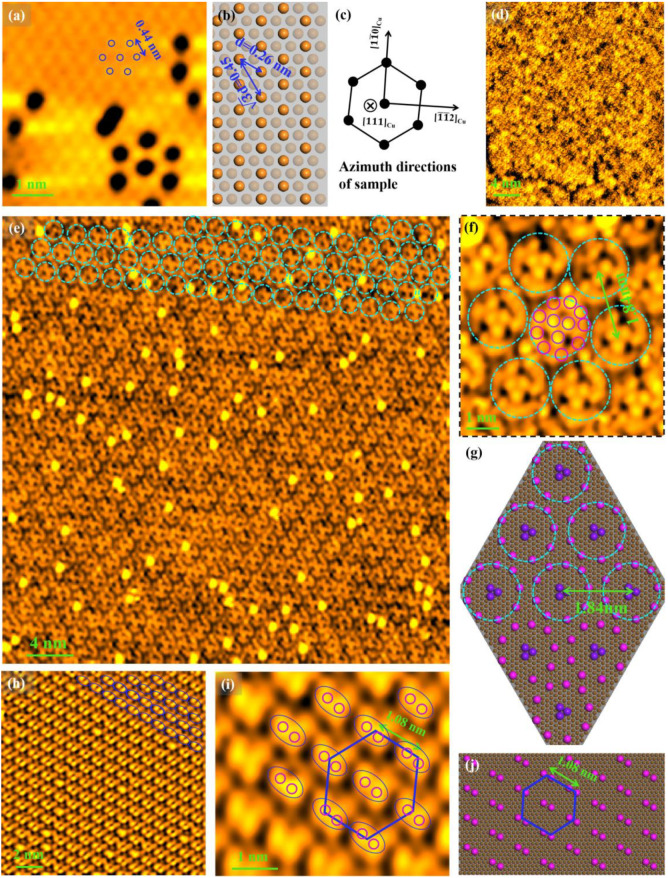
Surface structures of Process Ⅱ. (**a**) ($\sqrt{3}$ × $\sqrt{3}$ ) R30° reconstruction; (**b**) the atomic model of the Cu(111) surface, the double-blue arrows label the nearest (0.26 nm) and next-nearest (0.45 nm) atom distances, (**c**) [1$\bar{1}$ 0]Cu, [$\bar{1}\bar{1}$2]Cu, and [111]Cu azimuth directions of the sample according to the sample information; (**d**) a higher-resolution STM image from the triangle island region in Figure 1d; (**e**) a higher-resolution STM image of the smooth step terraces in Figure 1d, (**f**) an STM image with further higher resolution of the circular superstructure, (**g**) the corresponding atomic model in top view; (**h**) another higher-resolution STM image of the smooth step terraces in Figure 1d; (**i**) the STM image with further higher resolution of Figure 5h, the ellipses represent the double-atom structures, (**j**) the atomic model in top view. The blue hexagons of Figure 5i and j indicate the hexagonal symmetry of the superstructure. Here, the scanning bias voltage and current are equal to 0.5 V and 0.1 nA, respectively.

**Figure A1:**
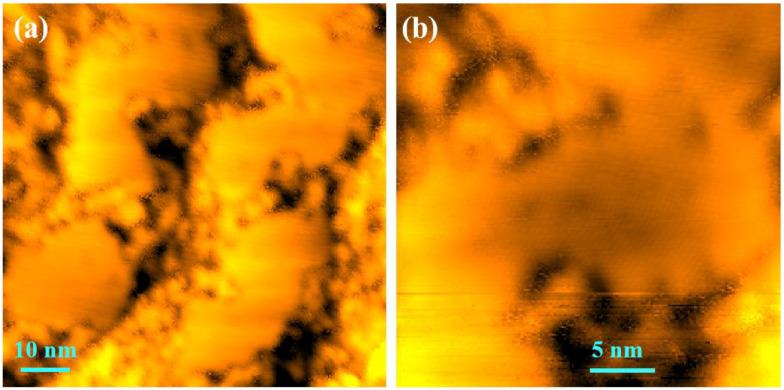
(**a**,**b**) High-resolution STM images from Process Ⅰ indicating disordered surface structures at this stage. Here, the scanning bias voltage and current are equal to 1 V and 0.1 nm, respectively.

We now discuss the details of the reconstructions and superstructures that emerge on the sample surface. Figure 1a demonstrates that the surface structure is almost fully disordered after annealing at 300 °C (Process Ⅰ), which is also confirmed by the corresponding high-resolution STM image Figure A1a,b (available in Appendix A). Intriguingly, there appear plenty of reconstructions with different structural features on the sample surface after annealing at 350 °C (Process Ⅱ). Figure 5a shows an atomic-resolved STM image of the ($\sqrt{3}$ × $\sqrt{3}$)R30° reconstruction of the smooth step terraces, in which the black holes directly reflect the presence of numerous point defects in such a reconstructed surface. Such reconstruction can be understood by the following comparative analysis of the experimental and theoretical data. Experimentally, the inter-atomic spacing (indicated by d_1_) is measured to be 0.44 nm [indicated by the blue double arrows in the STM image shown in Figure 5a], which is nearly identical to the next-nearest-neighbor distance of atoms (=0.45 nm) [indicated by the blue double arrows in the atomic model of Cu(111) surface in theory shown in Figure 5b]. This distance is equal to $\frac{\surd 6}{2}a$ for the bulk parameters (here, a = b = c = 0.362 nm) [29]. The nearest-neighbor distance of atoms [indicated by d and marked by the blue double arrows in the atomic model of Cu(111) surface] is equal to $\frac{\surd 2}{2}a$ (=0.26 nm). Notably, the ratio of the lattice constant after and before reconstruction is √3 (d_1_ = √3d) which is readily obtained from the above analysis. Figure 5c gives the schematic of the atomic structure in which three azimuths of [111]Cu, [120]Cu, and [1$\bar{1}$0]Cu reflecting the sample direction information are highlighted. As can be seen, a rotation of 30° occurs about the axis perpendicular to the Cu(111) surface between the structures before and after the reconstruction. Based on the above discussion, we conclude that a reconstruction of ($\sqrt{3}$ × $\sqrt{3}$)R30° has indeed occurred.

Figure 5d shows a higher-resolution STM image from the triangle island region in Figure 1d, revealing a fully disordered surface structure. Figure 5e corresponds to a higher-resolution STM image of the smooth step terraces in Figure 1d and shows the hexagonal superstructures (labeled by the dashed light blue circles), and the corresponding STM image with further higher resolution is shown in Figure 5f, showing that this superstructure consists of circular clusters (illustrated by the dashed light blue circles). The nearest-neighbor distance of circular clusters is measured to be 1.84 nm, as marked by the green double arrow [Figure 5f]. It can be seen more clearly that each cluster shows a unique circular structure with nine Cu atoms arranged circularly on the edge (labeled by the amaranth circles) and three atoms arranged triangularly in the center (labeled by the violet circles). According to the above results and discussion, the atomic model is drawn and shown in Figure 5g. Furthermore, we find the presence of another superstructure consisting of double atoms labeled by the blue ellipses in Figure 5h [another higher-resolution STM image of the smooth step terraces in Figure 1d] in some step terraces, which is probably the simplest superstructure. Figure 5i corresponds to the STM image with further higher resolution of Figure 5h, which more clearly shows the composition of “double-atom” structures of the superstructure, with the corresponding atomic model shown in Figure 5j. The distance between two adjacent double-atom structures [marked by the green double arrows in Figure 5i] can be easily calculated to be 1.08 nm. And the superstructure made of “double-atom” structures exhibits hexagonal symmetry, indicated by the blue hexagons shown in Figure 5i,j.

**Figure 6:**
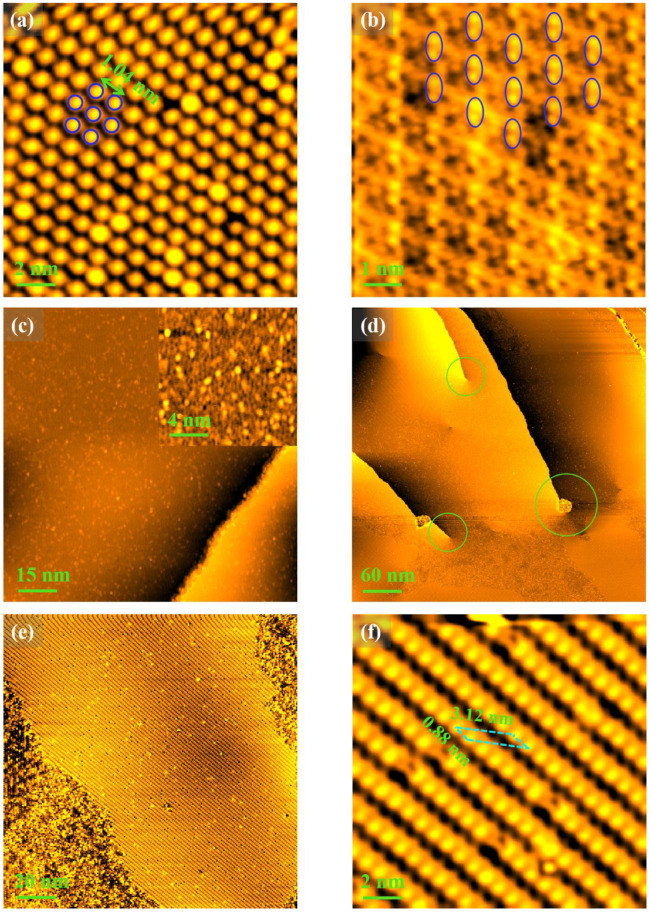
Surface structures from Process Ⅳ to Ⅵ. (**a**,**b**) High-resolution STM images (V = 0.5 V; I = 0.1 nA) from Process Ⅳ; the blue circles mark the Cu atoms (**a**) and the blue ellipses mark the “double-atom” structures (**b**); (**c**) STM image (V = 1 V; I = 0.1 nA) from Process Ⅴ sample, where the inset corresponds to the enlarged STM image, showing the disordered surface structure; (**d**) STM image (V = 1 V; I = 0.1 nA) from the Process Ⅵ sample, showing screw dislocations on the coexistence surface between disorder state and reconstruction, as labeled by the green circles; (**e**) a higher-resolution STM image showing a type of reconstruction with many defects on the disordered surface. (**f**) An atomic-resolution STM image of the reconstruction region. The scanning bias voltage and current are equal to 0.5 V and 0.1 nm, respectively.

After the annealing treatment of Process IV, some changes occur in the reconstruction and superstructure. It can be noted that a (4 × 4) reconstruction is present on the surface, which can be inferred from the distance between the two nearest atoms [1.04 nm (≈4a), indicated by the blue double arrow in a high-resolution STM image shown in Figure 6a]. Interestingly, the superstructure consisting of double atoms still exists in some localized regions, but the arrangement direction of the double atoms changes, as indicated by the blue ellipses in another high-resolution STM image shown in Figure 6b. For Process V, the surface structure becomes disordered, as illustrated by Figure 6c and the corresponding enlarged inset image. For Process Ⅵ, both disorder and order with reconstruction coexist on the surface, as shown in Figure 6d, from which the screw dislocations can be seen clearly, as marked by the green circles. Figure 6e is a higher-resolution STM image showing a type of reconstruction with many defects on the disordered surface. Figure 6f shows an atomic-resolution STM image of the reconstruction region, in which the basis lattice constants can be measured to be about 0.88 nm and 3.12 nm, respectively, as indicated by the blue dashed parallelogram. Comparing the basis direction with the sample orientation [Figure 5c], the rotation is calculated to be 8.5°. This result indicates that the surface structure belongs to a ($2\sqrt{3}$ × $7\sqrt{3}$)R8.5° reconstruction, as illustrated by the blue dashed parallelogram in Figure 6f.

### 3.3. Line Defects of Surface

**Figure 7:**
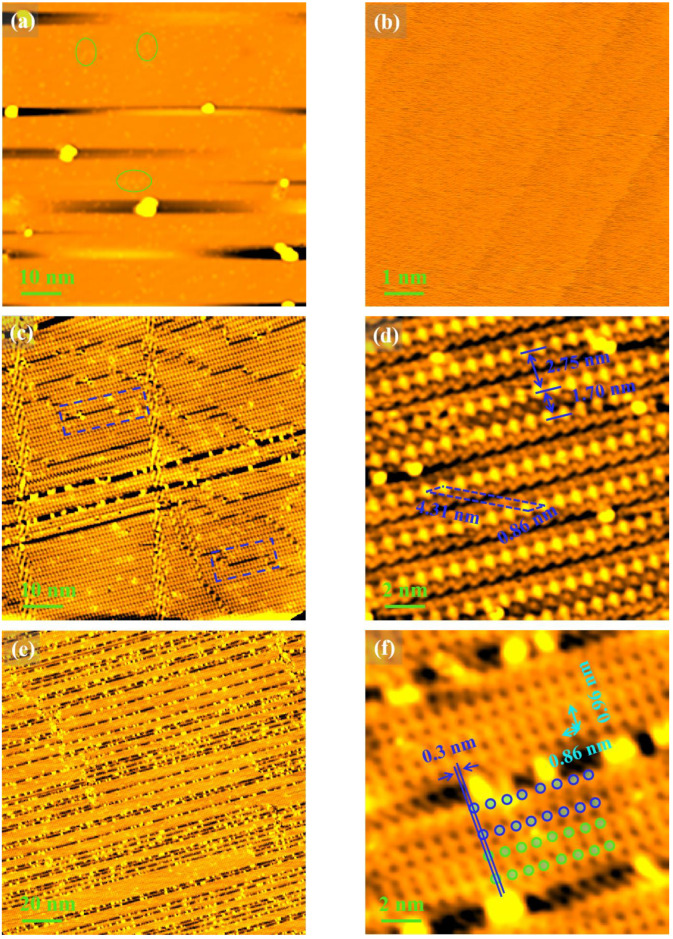
Surface structure from the samples in Process Ⅷ to Process Ⅹ. (**a**,**c**,**e**) The STM images (V = 1 V; I = 0.1 nA) of the sample surface after the treatment of Process Ⅷ, Process Ⅸ, and Process Ⅹ, respectively; (**b**,**d**,**f**) correspond to the higher-resolution STM images in (**a**,**c**,**e**), respectively. The blue and green circles in (**f**) mark the Cu atoms.

An STM image of the sample surface after the treatment of Process Ⅷ [Figure 7a] shows numerous adatoms labeled by the green circles. A higher-resolution STM image [Figure 7b] shows that the surface becomes disordered again. However, after annealing at 700 °C (Process Ⅸ), various degrees of linear deletion of atoms appear on the surface of the sample, as marked by the dashed blue rectangles in Figure 7c. From the corresponding atomically resolved STM image shown in Figure 7d, it is easy to calculate the basis lattice constants (0.86 nm and 4.31 nm) and rotation degree (13.5°), which accords with the structural feature of a ($2\sqrt{3}$ × $10\sqrt{3}$)R13.5° reconstruction. Notably, in some regions, the spacing of atomic rows is shortened to 1.70 nm from 2.75 nm, as shown by the double blue arrows in Figure 7d. The reason for this unusual condition might be the deletion of some atoms below the surface. When further increasing the annealing temperature to 760 °C, the number of both the missing and the absorbing atoms increase rapidly, as shown in Figure 7e. A major difference between Process Ⅸ [Figure 7c] and Process Ⅹ [Figure 7e] is that the deletion of atoms in the former case occurs in part of an atomic row, while in the latter case it occurs in a whole atomic row. These line defects are similar in character to those observed in borophene grown on an Ag(111) substrate [26]. To the best of our knowledge, this new surface phase on Cu(111) surface has not been previously reported in the literature. The formation of a large number of line defects leads to the formation of numerous parallel nanoribbons on the surface, as shown in Figure 7e, which is rather similar to the case of MoS_2_ ribbons grown on the Au(111) vicinal facet [32]. From a higher-resolution STM image showing the atomic structure of nanoribbons [Figure 7f], it can be seen that the lattice constants along the directions parallel and perpendicular to the atomic row are 0.86 nm and 0.96 nm, respectively, as marked by the double blue arrows. In addition, within the nanoribbon there exist two sets of lattices with a slight lateral shift (~0.3 nm) with respect to each other, as marked by the blue and green circles in Figure 7f. The spacing of atomic rows is shortened from 2.75 nm to 0.96 nm, which is also shown in Figure 7f. It is noticeable that the spacing of atomic rows is shortened for both cases presented in Figure 7d,f, but to a different degree. We believe that this stems from a stronger attraction force between the atoms when the removal of one or more atom rows from the adjacent nanoribbons is present.

## 4. Discussions

Next, we proceed to discuss the possible mechanism for the morphology and structure evolution of the Cu(111) surface. After Ar^+^ sputtering, a large number of atoms were adsorbed on the surface and many defects were formed. Under the appropriate annealing temperature conditions, these atoms can move into the lowest-energy positions through random motion [33], leading to smoother step terraces and an increasing trend of the step width and height with the increase of the temperature in the range of less than 600 °C. When the annealing temperature increases to above 600 °C, the desorption rate of surface atoms increases, and the step width and height decrease (due to the atoms close to the step edge detaching from the surface) with increasing temperature, as shown in Figure 4a. The reason for the appearance of the surface superstructure is that the adatoms tend to form some specialized 2D structures spontaneously through long-range electrostatic force. This behavior is similar to that of some metal atoms or 2D materials deposited on the Cu(111) substrates, in which cases some special superstructures also appear on the sample surfaces [34,35,36,37,38]. However, if the phenomenon of atomic desorption is more pronounced than that of the adsorption phenomenon, this type of surface structure is difficult to form, but there would emerge many line defects in the Cu(111) surface system, as shown in Figure 7e. Interestingly, in many oxide surface systems, this phenomenon of atomic desorption at high annealing temperatures would result in many special structures such as triangular or hexagonal holes on polar ZnO surface [39], quadrangle holes on CoO(001) [40], and polygon holes on TiO_2_(110) surfaces [41].

## 5. Conclusions

In this work, the surface morphology evolution of Cu(111) surface has been studied by low-temperature STM technique during ten periods of annealing processes with temperature oscillation (300 °C → 350 °C → 450 °C → 600 °C → 650 °C→ 720 °C → 420 °C → 420 °C → 700 °C → 760 °C). The results show that the step width and height exhibit first an increasing trend and then decrease with the increasing of the annealing temperature ranging from 300 °C to 720 °C. Surface morphologies exhibiting ideal surface features with large step width (>150 nm) and small step height (only one atomic layer thick) are successfully obtained at the temperature of 420 °C in the eighth annealing process. More importantly, two special superstructures (one consisting of circular clusters, and the other consisting of diatomic structures) and a new surface phase with many line defects were observed. We believe that our findings contribute to a better understanding of the commonly occurring superstructures in the 2D materials grown on Cu(111) surface.

## Figures and Tables

**Table 1:** Sample annealing parameters of the 10 processes.

	First Cycle		Second Cycle		Third Cycle	
	Temperature (°C)	Time (min)	Temperature (°C)	Time (min)	Temperature (°C)	Time (min)
Process Ⅰ	300	30	300	30		
Process Ⅱ	350	30	350	30	350	30
Process Ⅲ	450	3	450	3	450	3
Process Ⅳ	600	5	600	5	--	--
Process Ⅴ	650	3	650	3	--	--
Process Ⅵ	720	5	--	--	--	--
Process Ⅶ	420	5	420	5	420	5
Process Ⅷ	420	10	420	10	420	10
Process Ⅸ	700	5	700	5	720	5
Process Ⅹ	760	5	760	5	760	5
